# Hereditary gastric cancer checks its balance at the ATM: Broadening risk beyond CDH1

**DOI:** 10.1038/s41431-026-02127-5

**Published:** 2026-05-12

**Authors:** Paul C. Lott, Luis G. Carvajal-Carmona

**Affiliations:** 1 The Health Equity Leadership, Science, and Community Research Laboratory, Genome Center, University of California, Davis, USA; 2 Department of Biochemistry and Molecular Medicine, School of Medicine, University of California, Davis, USA

**Keywords:** Cancer genetics, Cancer genetics

The recently published article “Exome sequencing points to pathogenic *ATM* variants in gastric cancer” by Koebbe et al. [1] provides critical evidence and expands our understanding of *ATM*’s role in hereditary gastric cancer. Using germline exome data from a large multicenter study of early‑onset gastric cancer patients who were not selected for a known monogenic tumor syndrome and in whom no first‑degree relative with gastric cancer or other cancers suggestive of a hereditary cancer syndrome was reported, along with population‑scale data from gnomAD and the UK Biobank, the study found that pathogenic loss‑of‑function *ATM* variants were significantly enriched in gastric cancer patients [1].

These findings sharpen a timely question for human geneticists and clinicians alike: how should hereditary gastric cancer be conceptualized when reproducible genetic susceptibility extends beyond the small number of canonical high‑penetrance genes? This work invites further evaluation of current models of hereditary gastric cancer and its clinical management, which have primarily focused on a small set of highly penetrant genes and familial syndromes, most notably *CDH1* and *CTNNA1* [2]. It reinforces a growing body of evidence supporting a more complex genetic architecture in which moderate‑penetrance genes exert pleiotropic effects across multiple cancer types [3, 4].

## Re‑examining gastric cancer in a new light

While most gastric cancer is sporadic, approximately 10% of cases show familial clustering, of which only a small fraction is linked to established monogenic syndromes such as hereditary diffuse gastric cancer, gastric adenocarcinoma and proximal polyposis of the stomach, Lynch syndrome, familial adenomatous polyposis, juvenile polyposis syndrome, and Peutz‑Jeghers syndrome [2]. This means that most familial clustering in gastric cancer remains unexplained.

Koebbe et al. directly address this gap by adopting a genome‑wide, hypothesis‑free approach using whole‑exome sequencing and gene‑based burden testing, rather than focusing exclusively on predefined cancer‑predisposition panels [1]. This approach is particularly suited to detecting genes whose contribution to disease risk is distributed across many individually rare variants. The identification of *ATM*, enriched in their cohort and replicated in the UK Biobank, is methodologically and biologically notable, adding evidence that loss‑of‑function variants in *ATM* represent an important contributor to inherited gastric cancer risk in a subset of cases [1].

## ATM biology and multi‑organ cancer susceptibility

In 1995, mutations in *ATM* were confirmed to cause ataxia‑telangiectasia (A‑T), a rare recessive hereditary neurological disorder [5]. *ATM* is now recognized as a central regulator of the DNA‑damage response, coordinating cell‑cycle checkpoints, DNA repair, apoptosis, and responses to oxidative stress [6, 7]. In addition to the cancer predisposition observed in A‑T, heterozygous carriers of pathogenic *ATM* variants have been found to have an elevated susceptibility to multiple epithelial cancers, typically with a later age of onset than in classic high‑penetrance syndromes [8, 9].

Koebbe et al. strengthen the evidence that *ATM* is a key tumor‑suppressor by demonstrating enrichment of loss‑of‑function *ATM* variants in gastric cancer and replicating this association in the UK Biobank [1]. Importantly, the authors extend the clinical relevance of the finding by exploring the broader tumor spectrum in *ATM* loss‑of‑function carriers, observing enrichment across several solid tumor types, with prominent signals in upper gastrointestinal tract malignancies [1].

The strength of the association with gastric and esophageal cancers raises the question of shared environmental modifiers. These epithelial tissues are exposed to chronic inflammatory and oxidative stimuli, including smoking, dietary carcinogens, and *Helicobacter pylori* infection. Consistent with this possibility, recent evidence shows that *H. pylori* interacts with homologous recombination pathway genes to substantially increase gastric cancer risk, supporting a plausible framework in which *ATM* deficiency amplifies carcinogenic effects in exposure-rich contexts [10].

## Strengths, limitations, and open questions

The Koebbe et al. study has several notable strengths that enhance the credibility and impact of its findings. First, the combination of a multicenter early‑onset gastric cancer discovery cohort with independent replication in the UK Biobank provides a robust framework for discovery and validation [1]. Second, genome‑wide gene‑burden testing captures the cumulative effect of rare loss‑of‑function variants within a gene, an especially appropriate strategy for genes such as *ATM*, where individual pathogenic variants are exceedingly rare [1]. Third, the multi‑cancer analyses provide valuable biological context, situating gastric cancer risk within a broader spectrum of tumor predisposition relevant to genetic counseling [1].

These strengths notwithstanding, important limitations remain. Although this is one of the largest early‑onset gastric cancer exome‑sequencing efforts to date, the sample size is still insufficient to estimate penetrance precisely or determine age‑specific absolute risks for *ATM* carriers [1]. Differences in ascertainment and reliance on population controls can also limit direct clinical extrapolation.

Moreover, while the statistical association between *ATM* and gastric cancer is compelling, functional validation specific to gastric tumorigenesis remains limited. Key unanswered questions include tissue‑specific mechanisms, the presence and nature of somatic second hits, and whether gastric tumors arising in *ATM* carriers show consistent molecular features that would strengthen causal inference and inform clinical management [1].

## Broader implications for cancer genetics

The identification of *ATM* as a reproducible gastric cancer susceptibility gene marks a meaningful advance in cancer genetics. This study adds to a growing body of literature showing that moderate‑risk genes play an important role in the etiology of gastric cancer, challenging paradigms that focus almost exclusively on high‑penetrance monogenic syndromes [3, 4].

From a clinical perspective, Koebbe et al. extend and independently validate the conclusions of our recent international gastric cancer genetics study. Together [4], these data indicate that when monogenic gastric cancer is suspected, especially in early‑onset cases, families with clustering of cancers within the broader *ATM* spectrum, or tumors with non‑diffuse histology, *ATM* (along with other pathway related genes [10, 11]) should be considered alongside, and beyond, the canonical gastric cancer genes *CDH1* and *CTNNA1*.

## Priorities for future investigation

Future priorities include large prospective cohorts to quantify penetrance, integrated germline–somatic analyses to clarify tumor evolution in the context of *ATM* deficiency, and deliberate inclusion of diverse populations to ensure generalizability. This emphasis is particularly important given that non‑European populations experience a disproportionate burden of gastric cancer, including higher incidence, later stage at diagnosis, poorer outcomes, and persistent underrepresentation in cancer genetic and genomic studies [12, 13]. Ultimately, incorporating *ATM* and other moderate‑penetrance genes into the framework for gastric cancer susceptibility could improve the identification of at‑risk individuals and deepen understanding of the biological and social determinants of this challenging disease.
